# The Case against Maintenance Charges

**Published:** 1921-03-26

**Authors:** 


### THE CASE AGAINST MAINTENANCE CHARGES.

The advisability of a maintenance charge for in-patients was again considered by the Governors of the General Hospital, Birmingham, on March 16. It will be remembered that the question was first raised in July of last year, but the operation of the scheme was postponed on the strong recommendation of the representatives of employees of several of the large local firms to enable the promoters of the General Hospital "League" to make an appeal to sources hitherto untapped. Six months, it was suggested, would prove a good test of the possibilities of the League, but on the recommendation of the Board of Management a further postponement of the maintenance charge for another three months has been agreed to.

Although the hospital was fortunate enough to receive a grant of £17,800 from the National Relief Fund, the board has found it necessary to sell £80,000 worth of investments at a loss of over 40 per cent., and the year's working shows an excess of expenditure over ordinary income of £30,000.

Among certain classes there is a growing opinion in favour of a maintenance charge as one way to solve the present financial difficulties, but the effect of its introduction in the case of a large general hospital serving an industrial area of definite limits would probably much decrease, if not destroy, voluntary contributions from large factories. Many of these are subscribing yearly more than four times the total annual amount chargeable under a maintenance scheme. There is every reason to believe that such firms would at once discontinue their subscriptions and pay simply the amount chargeable for the number of patients treated from their separate factories.

In these circumstances it seems that to start any maintenance scheme would be harmful. Apparently the only certain way out of the difficulty is a renewed effort to get increased subscriptions from workpeople and others, in addition, of course, to tap more thoroughly the non-subscribing public.

In moving the adoption of the report and statement of accounts the Bishop of Birmingham (President) said he was afraid that the younger generation is not being educated in their responsibility toward charity: the illustrious example of their elders is not being followed with much enthusiasm. He appealed to all present, and also to the representatives of the Press, for help that the future citizens of the city might live (and work) to see the hospitals of the country placed on a sound basis.

